# Urban contact patterns shape respiratory syncytial virus epidemics with implications for vaccination

**DOI:** 10.1126/sciadv.ady5457

**Published:** 2025-11-26

**Authors:** Presley Kimball, Jean-Sebastien Casalegno, Pamela P. Martinez, Ayesha S. Mahmud, Bjorn Sandstede, Rachel E. Baker

**Affiliations:** ^1^ Division of Applied Mathematics, Brown University, Providence, RI, USA.; ^2^ Hospices Civils de Lyon, Hôpital de la Croix-Rousse, Centre de Biologie Nord, Institut des Agents Infectieux, Laboratoire de Virologie, Lyon, France.; ^3^ Department of Microbiology, University of Illinois Urbana-Champaign, Champaign, IL, USA.; ^4^ Department of Statistics, University of Illinois Urbana-Champaign, Champaign, IL, USA.; ^5^ Department of Demography, University of California, Berkeley, CA, USA.; ^6^ Department of Epidemiology, Brown University, Providence, RI, USA.; ^7^ Institute at Brown for Environment and Society, Brown University, Providence, RI, USA.

## Abstract

Urban environments may alter the landscape of disease transmission with implications for control. Yet, it is unclear whether urban-rural differences exist in the dynamics of childhood respiratory diseases, given specific mixing patterns in younger age groups. Here, we leverage county-level data on respiratory syncytial virus (RSV) from the United States to reveal an urban-rural gradient in both the intensity and age structure of the RSV epidemic, where urban locations experience more prolonged epidemics with higher burden in infants (under 1 year of age). We develop a mechanistic epidemiological model to show that these differences can be explained by daycare utilization rates in children under 5. Using our model to consider control measures, we find that expanding seasonal immunization access in urban and rural areas may limit the risk of off season RSV epidemics.

## Teaser

The age structure and intensity of RSV outbreaks differ across urban and rural locations.

## INTRODUCTION

Respiratory syncytial virus (RSV) is a leading cause of lower respiratory tract infections in young infants, accounting for approximately 80,000 hospitalizations in the US annually for children under 5 (*1*). RSV dynamics vary substantially by location, both globally and within the US (*2*). Outbreak patterns range from biennial (a large epidemic peak every 2 years) in northern US states to annual or year-round in southern parts of the US and the tropics (*3*, *4*). Climate factors are expected to drive these regional differences in outbreak patterns with evidence suggesting that locations with a larger seasonal range in climate may experience more intense outbreaks (*4*). However, it remains unclear whether other factors, such as population structure and contact patterns, may play a role in governing RSV transmission, as has been suggested for other pathogens (*5*, *6*). Characterizing the drivers of RSV dynamics may be particularly important to optimize control policies given the recent introduction of a maternal vaccine and an injectable monoclonal antibody for infants for the 2023–2024 RSV season in the US (*7*, *8*). Both of these approaches are passive immunization strategies that confer a limited window of protection on infants. Hence, the efficacy of these measures in terms of reduction in RSV hospitalizations, and RSV epidemic peak size, may depend on the case age structure and local RSV seasonality.

Urban environments are expected to play a unique role in disease transmission, both in the present and in the future, as human populations increasingly inhabit dense urban environments (*9*, *10*). High population density in urban locations may lead to increased contacts, while mobility and connectivity between urban locations may increase exposure (*11*, *12*). At the same time, transmission of respiratory pathogens is often modeled as frequency dependent, reflecting proposed stable per-capita contact rates even as population density increases (*13*, *14*). Preliminary evidence for respiratory pathogens suggest that outbreaks in urban environments are prolonged with less distinct epidemic “peaks” (*5*, *15*). For example, influenza was found to have less intense outbreaks in urban settings—where intensity is defined on the basis of the Shannon entropy of the annual incidence distribution (*5*). Similarly, outbreaks of COVID-19 were found to be more spread out over time in urban environments (*15*). Yet, it is unclear whether these patterns can be generalized to all respiratory pathogens; in particular, it remains uncertain whether the urban environment should differentially affect transmission for a childhood diseases such as RSV. Crucially, it is unclear whether urban-rural differences in epidemic intensity alter the age structure of epidemics, which remains important for diseases such as RSV where early first infection may increase vulnerability to severe disease (*16*).

Here, we leverage US county-level data on RSV hospitalizations by age group to explore the role of urbanization on the intensity and age structure of RSV epidemics. We develop an age-structured RSV model which we fit to data to explore the effect of different urban-rural contact patterns on RSV outbreak dynamics. Childcare settings have been shown to be an effective environment for RSV transmission (*17*). Using data on daycare enrollment from the Survey of Income and Program Participation (SIPP), a survey distributed by the US Census Bureau, we explore whether increased daycare enrollment in urban areas can explain urban-rural differences in outbreak intensity and age structure. We then use our model to consider the implications of RSV immunizations for outbreak dynamics across the urban-rural gradient.

## RESULTS

### Urban-rural differences in outbreak intensity and age structure

**Fig. 1.**
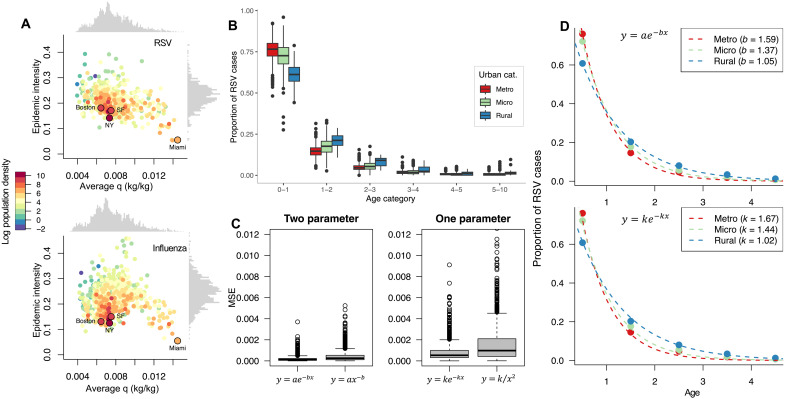
Urbanization affects RSV age distribution and outbreak intensity. (**A**) County-level mean specific humidity “q” and epidemic intensity for RSV (top) and influenza (bottom). Color gradient is log population density (capita per square kilometer). (**B**) The age distribution of RSV for age groups under 10 conditioned on urbanization category. (**C**) Mean squared error comparison of potential models for the age distribution of RSV. (**D**) Two models of the RSV age distribution demonstrating the difference of curvature due to urbanization: $y={\mathrm{ae}}^{-\mathrm{bx}}$ (top) and $y={\mathrm{ke}}^{-\mathrm{kx}}$ (bottom). Steeper curves are due to higher urban categorization which corresponds to higher values of *b* and *k*, respectively.

Figure 1A shows the correlation between climate (average specific humidity), urbanization (log population density), and epidemic intensity for both RSV (top plot) and influenza (lower plot) where each point is based on hospitalization data at the county level. Epidemic intensity is calculated using the Shannon entropy of the average annual incidence distribution and scaled so that the intensity metric is 1 if all cases occur within 1 week of the year and zero if cases are exactly evenly distributed throughout the year (see Materials and Methods). We find a significant positive effect of both climate and population density on RSV epidemic intensity (fig. S1). As observed for influenza, the color gradient reveals that locations with a high population density experience lower intensity RSV epidemics (*5*).

We also explore the effect of urbanization on the age structure of RSV outbreaks. Figure 1B shows the average age distribution of RSV for individuals living in three urbanization categories based on definitions given by the United States Office of Management and Budget (OMB) where “metro” refers to counties with a population greater than 50,000, “micro” consists of counties with a population of 10,000 and 50,000, and counties designated as “rural” are those with any population less than 10,000 (*18*). We partitioned the dataset based on these categories using the county-level population in the 2010 US Census (*19*). In metro counties, we find a higher average proportion of hospitalizations of infants under 1 year old compared with micro and rural counties (see table S1). In contrast, metro and micro counties have on average a smaller proportion of RSV cases in the 1- to 2-year-old age category than rural counties (see table S2). This indicates that children may have a higher chance of contracting RSV at an earlier age in more urban environments [analysis of variance (ANOVA) testing provided statistical evidence of differences (α=0.05) in the mean proportion of RSV cases for age groups under 10 years old when conditioned by urban categorization (see table S3 for values)]. We find that this difference in the age distribution of RSV due to urbanization is not seen in the population-level age distribution (fig. S2 and table S4), as found in 2010 county-level data from the US Census Bureau (*20*).

To better capture rural-urban differences in age structure, we fit four parameterized curves: $y={\mathrm{ae}}^{-\mathrm{bx}}$, $y={\mathrm{ax}}^{-b}$, $y={\mathrm{ke}}^{-\mathrm{kx}}$, and $y=\frac{k}{x^{2}}$ to age-structured data for each US county (see fig. S3 for examples of the fitted curves). We then use the mean squared error to determine the best fit curves across all locations (Fig. 1C). We find that $y={\mathrm{ae}}^{-\mathrm{bx}}$ is the best fitting two-parameter curve, and the exponential probability density function, $y={\mathrm{ke}}^{-\mathrm{kx}}$, is the best fitting one-parameter curve. Differences in the distributions’ rate of change are encapsulated within parameters *b* and *k*, respectively. We find that fitted values of *b* and *k* are higher in urban (metro) counties than rural counties (Fig. 1D). The differences in parameters across urbanization category are also statistically significant (see table S5).

**Fig. 2.**
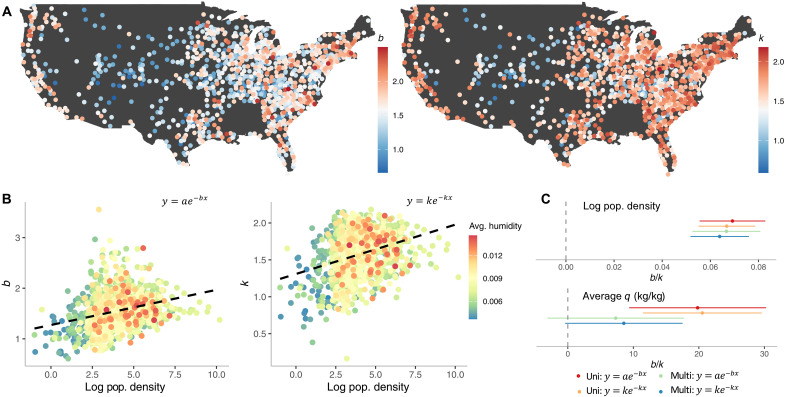
Urbanization correlates with RSV hospitalization age structure. (**A**) Map of the United States colored by the value of *b* (left) and *k* (right). (**B**) Correlation plots between the log of population density and parameter *b* from $y={\mathrm{ae}}^{-\mathrm{bx}}$ (left) and *k* from $y={\mathrm{ke}}^{-\mathrm{kx}}$ (right), colored by the average historic humidity. (**C**) Coefficient values for separate linear regression of log of population density (top) and average historic humidity (bottom) on age structure shape parameters *b* and *k*.

Figure 2A shows how fitted *k* and *b* parameters, our measure of age structure, vary spatially (Fig. 2A) across the US. In rural areas, like the Midwest, we observe lower values of *b* and *k*, whereas we observe higher values of the parameters mainly along the coasts. The spatial consistency between the *k* and *b* parameters provides support for the observed pattern of increased RSV burden at younger ages in certain regions. Figure 2B shows the plotted association between population density, climate, and age structure. On average, higher population density is correlated with higher *b* and *k* values representing a higher proportional burden in younger age groups. Univariate and multivariate linear regression shows a significant positive association between the age distribution parameters and log population density (Fig. 2C). Specific humidity, a hypothesized climate driver of seasonal RSV transmission (*4*), showed a significant association in the univariate regression model but was not found to be a significant determinant of age structure in the multivariate model (Fig. 2C and tables S6 and S7), implying that the univariate effect might be confounded by the association with population density. To explore potential nonlinear relationships, we extended our analysis using generalized additive models, which revealed that the shape parameters are more strongly influenced by log population density than by average specific humidity (see Supplementary Text and fig. S4).

### Daycare usage explains urban-rural differences in RSV outbreak patterns

**Fig. 3.**
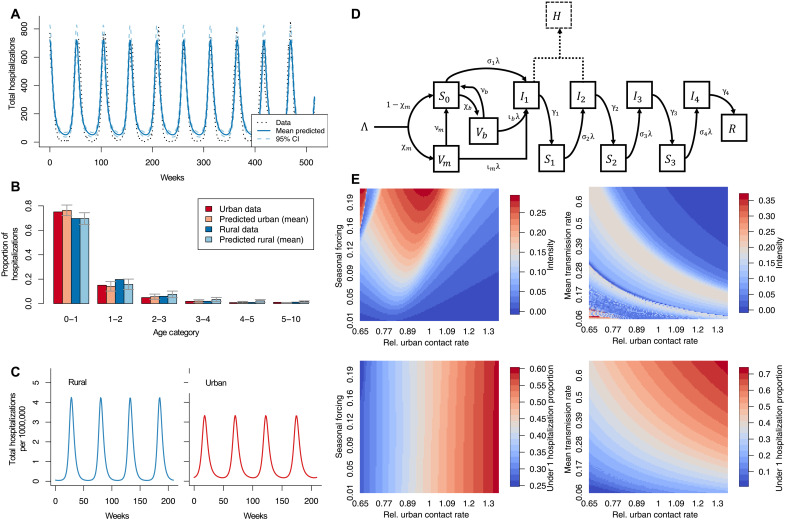
Modeled impact of urbanization on RSV dynamics. (**A**) Model fit to RSV cases in Texas over a 10-year period. The 95% confidence intervals (CI) were generated using the best 500 of 10,000 parameters generated during model fitting. (**B**) Comparison between data and model prediction of RSV age distribution (by age category in years) due to urbanization of Texas generated by using a 1.15 relative urban contact rate amplification for the urban category. (**C**) Standardized modeled population dynamics in rural and urban environment over 4 years. Urban and rural environments modeled using 1.15 relative urban contact rate amplification for the urban category. (**D**) Model diagram of infant compartment of ODE model of RSV adapted from (*21*). Diagram generalizes to other age categories by setting Λ, $\chi_{b}$, and $\chi_{m}$ to zero. For simplicity, the diagram also omits flows due to death and flows between age categories due to aging. (**E**) Heatmaps of intensity (top) and proportion under 1 hospitalizations (bottom) under a range of relative urban contact rates, seasonal forcing, and mean transmission.

Our results suggest that urbanization, specifically population density, is an important predictor of both the intensity and age structure of RSV epidemics. We find that more urban areas tend to have more protracted, lower intensity outbreaks, with a higher burden in the under 1 year age group when compared to rural areas. To test possible mechanistic drivers of these patterns, we develop an age-stratified SIRS (susceptible-infectious-recovered-susceptible) model for RSV, modified from prior work (*3*, *21*) (see Materials and Methods for model details, Fig. 3D for the model diagram, and table S8 for model parameters). The model accounts for differing risks of infection and hospitalization based on age and stage of infection. In particular, the model assumes that the hospitalization rate is highest for infants under age one and monotonically decreases with age (see Materials and Methods for full discussion). We fit our model to state-level data over a 10-year period in Texas using Latin hypercube sampling (see Fig. 3A). We select Texas due to its size, which enables representation of all urban-categorized county types, as well as stability in epidemic dynamics due to its climate and location (*4*). Because of limited data from rural locations and the relatively low population size in micro counties, we group rural and micro counties together and categorize them as rural for comparison to metro locations, which we designate as “urban.”

The matrix of contact rates between age groups in our model is based on prior estimates for the US population as a whole (*22*). We allow urbanization to enter the model by including an “urban contact rate” parameter which amplifies contact rates for young children in urban environments. Our decision to include the urban contact rate parameter is based on an analysis of data from the SIPP from the US Census Bureau. We calculate the proportion of parents with children under 5 who report the child to be in daycare (further delineation by age group was not available). We find a 26.9% daycare usage rate in metro (urban) areas versus a 23.4% daycare usage rate in nonmetro (rural) areas. On the basis of these numbers, we adjust the relative urban contact rate assuming a 1.15 relative increase (26.9/23.4 = 1.15) in the contact rate in urban environments.

Figure 3B compares our model predictions to the observed data for Texas in terms of proportion hospitalizations by age category. We find that the boosted contact rate among younger age groups in metro areas leads to an increase of hospitalizations of infants under 1 year old in these locations, as reflected in the observations (including boosted contact rates across all age groups does not affect our results; see fig. S5). This is due to the amplified contact rate for young children in urban areas, and infants under 1 year old are at higher risk for hospitalization than older age groups, although this risk is the same in both urban and rural counties. In contrast, the reduced contact rate in nonmetro areas leads to a relative increase in hospitalizations in the 1- to 2-year age group when compared to metro locations. The numbers from Texas represent an average across many nonmetro and metro counties, and hence overall differences between nonmetro and metro proportions remain modest. Nevertheless, our model is able to capture this mean rural-urban difference.

In Fig. 3E, we explore the effects of varying the relative urban contact rate, mean transmission rate, and seasonal forcing on RSV epidemic intensity and age structure (proportion of hospitalizations in the under age 1 category). We find that increasing the relative urban contact rate leads to lower intensity RSV outbreaks (example trajectories are shown in Fig. 3C) and a greater proportion of hospitalizations of under 1-year-old infants, matching our observations in Fig. 1 (see fig. S6 for an exploration of nonlinearities in Fig. 3E). Further, we find that increasing the amplitude of seasonal forcing leads to more intense outbreaks on average but does not appear to affect the age structure of outbreaks. This is supported by the multivariate regression results on the curve parameters which showed that, controlling for population density, specific humidity was not correlated with age structure (see tables S6 and S7).

### Implications of RSV immunization for outbreak dynamics and age structure

Two immunizations have been approved in the United States to prevent severe RSV disease in young children, starting in the 2023–2024 RSV season: a monoclonal antibody infusion to be administered in infants in the first year of life, and a maternal vaccine, administered to pregnant women between 32 and 36 weeks of pregnancy during September through January (*1*). The maternal immunization is estimated to reduce risk of severe RSV by 57%, while the infant immunization is estimated to reduce risk of severe RSV by 81% (*23*). Immunizations are modeled as reducing the risk of transmission to vaccinated individuals, with protection waning over time, following prior work (*24*).

**Fig. 4.**
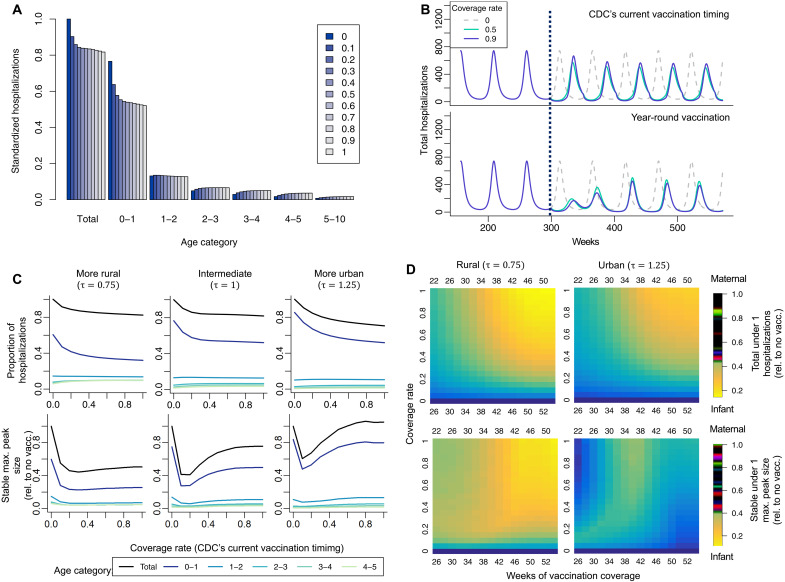
Modeled impact of immunization across urban-rural gradient. (**A**) Reduction in RSV cases across age categories (years) based on the immunization coverage rate. (**B**) Change in dynamics due to immunization timing and varying coverage rates. (**C**) The effect of increased coverage rate on proportion of hospitalizations and stable maximum peak size. Results are shown for three levels of urbanization, each corresponding to different values of the relative urban contact rate parameter, τ. (**D**) Heatmap of the effect of increasing weeks of maternal and infant vaccination coverage, relative to the CDC’s current seasonal vaccination timing (leftmost column), on dynamics for infants under 1 year old, measured by total hospitalizations and stable maximum peak size.

In Fig. 4A, we estimate the effect of changing immunization coverage (for both the maternal vaccine and infant antibody infusion) on hospitalizations by age category. The effect is shown relative to zero immunization coverage so that total hospitalizations in the zero immunization simulation are equal to one. As expected, we find that increased immunization coverage leads to a reduction in total RSV hospitalizations. Moreover, we find that children under age 1 experience the greatest reduction in RSV burden, while we predict a modest increase in hospitalizations for certain older age classes.

We also explore the implications of the current immunization schedule for RSV outbreak dynamics. In Fig. 4B, we find that, across different coverage rates, the current seasonal US Centers for Disease Control and Prevention (CDC) immunization schedule (the infant immunization is administered 26 weeks out of the year, and the maternal vaccine is administered 22 weeks out of the year) may lead to a shift in timing of the RSV epidemic to the summer months. Given the public health implications of this ongoing outbreak, we explore alternate immunization schedules. We find that offering year-round immunizations may reduce the risk of a summertime outbreak, even in a location, such as Texas, where RSV outbreaks have a typical wintertime seasonality.

We investigate the effect of changing the immunization coverage rate on stable RSV peak size (observed after 15 years to account for transient dynamics) and under 1 hospitalizations for urban and rural locations in Fig. 4C (note that in this figure, hospitalizations and peak size are shown relative to the zero coverage result for each location type; see fig. S7). We find that increasing the immunization coverage rate in urban counties leads to a steeper decline in RSV hospitalizations when compared to rural areas. This is because immunizations benefit very young children (age 0 to 1), and the proportion infected in this age group is higher in urban areas. Both rural and urban areas experienced a predicted decline and then increase in RSV stable peak size as the coverage rate increases. We find that as vaccine coverage increases, the RSV outbreaks more firmly shifts to the summer as there is less ongoing transmission while seasonal immunization is in effect (see fig. S8); this effect is most strong in urban locations as higher transmission allows outbreaks to rebound more quickly during the period without vaccine coverage (for further discussion, see Supplementary Text).

In Fig. 4D, we show the implications of increasing both immunization coverage and the number of weeks in the year that both immunizations are offered in both urban and rural locations. We find that offering immunizations year-round leads to the greatest reduction in hospitalizations and stable peak size in both rural and urban locations. In rural locations, the reduction in peak size and reduction in hospitalizations are minimized more quickly under expanding seasonal coverage than in urban areas. In general, rural areas experience a greater reduction in under 1 hospitalizations for seasonal expansion of immunization access when compared to urban areas. This is due to the proportion of hospitalizations in the under 1 age group being higher in urban areas in the case with and without immunization. In contrast, urban areas have much larger total population numbers than rural areas, meaning that a smaller percentage reduction in hospitalizations could still affect a large number of infants. In addition, the higher force of infection in urban areas results in a stronger summertime outbreak under seasonal vaccination: Year-round vaccination coverage minimizes the risk of this outbreak (Fig. 4D and fig. S8).

## DISCUSSION

Our analysis reveals an urban-rural gradient in the age structure and intensity of RSV epidemics. Using our mechanistic RSV model, we find that increased contact rate between children, potentially driven by increased daycare usage in urban areas, can explain both changes to age structure and intensity. In urban areas, higher contact rates lead to a higher proportion of hospitalizations in the under 1 age group and a more prolonged, lower intensity RSV outbreak. In addition, our results suggest that while epidemic intensity is influenced by climate-driven changes to transmission seasonality as well as population factors, climate factors do not appear to affect the age structure of the outbreak when we account for urbanization factors (Figs. 2C and 3E and fig. S4, assuming changes to seasonal forcing proxy for different climate conditions).

We modeled how RSV outbreak dynamics may change under the implementation of two immunizations. We find that any level of additional vaccine coverage lowers RSV hospitalizations; however, the current seasonal implementation of immunizations that is recommended by CDC guidelines may increase the risk of a summertime RSV outbreak. To fully minimize the risk of a large seasonal outbreak, our results suggest that access to RSV immunizations be provided year round. Year-round immunization lowers the size of annual peak outbreaks, while the dynamics exhibit consistent, annual stability. We note that because of current efficacy results for RSV immunizations, year-round immunization access does not eliminate the RSV epidemic. This is supported by prior modeling work in Australia that estimates a 50% reduction in RSV hospitalization for young infants and a 4% reduction in total infections across age groups (*25*).

There are a few limitations in our study. Our immunization results are based on a model fitted to data from Texas. Texas has regular seasonal outbreaks of RSV and a standard wintertime outbreak timing; hence, we expect our immunization recommendations to be broadly applicable to other locations. However, future work could aim to target the timing of immunizations to the dynamics within each US state: Locations with biennial outbreaks or with persistent year-round cases may be differentially affected by the current CDC vaccination schedule. Linking transmission seasonality to climate drivers could improve the generalizability of our model to other locations. Second, our fitted model to Texas does not fully capture the depth of the incidence trough during summer months which may be due to changes in testing and reporting outside of the typical RSV season. To assess whether this affects our vaccination modeling results, we simulated the Texas model including a seasonal step function to force a reduction in cases during the summer months (fig. S9). We find that the shift in RSV outbreak timing is still observed after introducing immunization, using this alternate model. Again, we find that switching from a seasonal to year-round vaccination program mitigates this outbreak. Last, our model assumes that both immunizations reduce transmission and the risk of severe disease, following prior work (*24*). However, it is possible that immunizations reduce the risk of severe disease without affecting transmission, in which case RSV dynamics would not be disrupted by seasonal immunization. In this scenario, we still expect greater net reductions in hospitalizations in urban areas due to the higher proportion of under 1 children in these locations; however, we would not expect a change to RSV peak size following vaccine introduction.

More broadly, our work highlights a role for population structure in determining the dynamics of a childhood disease, RSV, as has been observed for other respiratory pathogens that circulate among adults. In particular, we find a relationship between urbanization and age structure which has implications for control: Such a relationship may exist for other pathogens and could be explored in future work. As increasing numbers of individuals move to urban areas, and changes to the workforce increase daycare utilization, understanding the role of these factors in determining outbreaks is important for understanding future risk. Furthermore, the seasonal timing of immunization programs might differentially affect outcomes across geographies as determined by climate and population structure. Increased efforts to synthesize these multiple drivers of disease burden may help improve targeting of these programs both within the US and abroad.

## MATERIALS AND METHODS

All analyses and computations were performed in R v4.3.2 (*26*). See Supplementary Text for packages used and versions.

### RSV hospitalization data

Data on RSV hospitalizations come from the State Inpatient Databases (SIDs) of the Healthcare Cost and Utilization Project (HCUP) maintained by the Agency for Healthcare Research and Quality. SID data represents 96% of community hospital inpatient discharges from reporting states, although not all states have opted in to report data to HCUP. RSV diagnosis is based on International Classification of Diseases 9th revision, Clinical Modification code for RSV (079.6, 466.11, and 480.1). We cleaned the data to remove sparse observations using a threshold number of 50 total reported cases total. We also remove data before 1998 due to a change in reporting codes for RSV that occurred in 1996. After cleaning, the data consist of 1145 counties. For statistical analysis, we couple our RSV metrics with county-level specific humidity data from the North America Regional Reanalysis gridded dataset, produced by the National Centers for Environmental Prediction (*27*).

### RSV hospitalization data analysis

We first performed statistical tests to assess differences in the proportion of RSV cases across each age category based on urban categorization. To define the urbanization categories, we use the metric from the United States OMB (*18*). Under this metric, metro populations are those with a population more than 50,000, micro populations are those with population between 10,000 and 50,000, and rural populations are those under 10,000. Because of limited representation of rural counties at the state level, the categorization becomes binary by redefining metro counties to be urban, and we group micro and rural counties to be relabeled as rural counties. This binary redefinition is also defined by the OMB (*18*).

#### Measuring the age distribution of RSV

We fit four parameterized curves to measure the differences in the RSV age distribution of each location. We analyzed results for the following curves: $y={\mathrm{ae}}^{-\mathrm{bx}}$, $y={\mathrm{ax}}^{-b}$, $y={\mathrm{ke}}^{-\mathrm{kx}}$, and $y=\frac{k}{x^{2}}$. Note that $y={\mathrm{ke}}^{-\mathrm{kx}}$ is the equation for the exponential probability density function. We fit each of these curves to each location in our cleaned dataset using nonlinear least squares regression. We also fit each curve more generally to each urbanization category by grouping across counties. We evaluated the error of each of these curves using the mean squared error values generated during fitting. We proceeded with more in depth analysis by selecting the best fitting one and two parameter models.

We then conducted mixed and simple linear regression for predicting each location fitted values of *b* and *k* for $y={\mathrm{ae}}^{-\mathrm{bx}}$ and $y={\mathrm{ke}}^{-\mathrm{kx}}$, respectively, using log of population density and average humidity. Furthermore, we performed ANOVA tests followed by Tukey’s post hoc tests for pairwise differences in parameter values based on urbanization category among all age groups under 10. Last, we conducted ANOVA tests on the population-level age distribution from the 2010 US Census Bureau (*20*) to assess whether differences in the RSV age distribution could be explained by underlying population-level differences.

### ODE model description

To simulate the results we found in data analysis and predict possible vaccination strategies, we constructed an age-structured RSV model following the direction of (*21*). Let A denote the age categories where

$$A=\{\left[0,1\right),\left[1,2\right),\ldots ,\left[4,5\right),\left[5,10\right),\left[10,15\right),\ldots ,\left[70,75\right),\left[75,\infty \right)\}$$

where $\left[75,\infty \right)$ represents ages above 75 and ∣A∣=20. We define

$$S^{j}=\left\{S_{0}^{j},S_{1}^{j},S_{2}^{j},S_{3}^{j},I_{1}^{j},I_{2}^{j},I_{3}^{j},I_{4}^{j},R^{j},V_{m}^{j},V_{b}^{j}\right\}$$

as the set of states such that for age group *j*, $S_{i}^{j}$ represents susceptible after receiving the infection *i* times, $I_{i}^{j}$ represents infected during their *i*th infection, $R^{j}$ is recovered, and $V_{m}^{j}$ and $V_{b}^{j}$ represent vaccinated individuals who received a maternal vaccine or an infant immunization, respectively. The entire state space consists of the collection of $S^{j}$ across all age classes for j∈A. Our model uses four infection compartments, representing the first four RSV infections, following the direction of (*21*). In their study, authors integrate empirical evidence showing that pediatric RSV reinfections are common and that immunity is not sterilizing (*28*, *29*). After the fourth infection compartment, individuals flow to a recovered compartment due to the assumptions that both the hospitalization rate decreases with age and number of infections and that the risk of infection decreases with the number of infections (*21*). Under these assumptions, infections that occur after the fourth are considered negligible in contributing to the force of infection or the hospitalization rate.

The birth rate is represented as Λ, and we use the birth rate estimate for Texas (*19*, *30*). We represent the death rate as μ. Since our model focuses on a childhood infection, the death rate does not change with age, and the death rate also does not change with RSV infection. An individual is believed to acquire increased immunity with more RSV infections (*21*), so we let $\sigma_{i}$ estimate the risk of the *i*th RSV infection for a susceptible individual. $\gamma_{i}$ represents the recovery rate of the *i*th RSV infection for each individual. We control for vaccination in our model through coverage rates $\chi_{m}$ and $\chi_{b}$, waning vaccination rates $\nu_{m}$ and $\nu_{b}$, and lower risk of infection $\iota_{m}$ and $\iota_{b}$ for the maternal and infant vaccinations, respectively. Note that $\chi_{m}$ and $\chi_{b}$ are 0 when $j\in A\backslash \{\left[0,1\right)\}$ since we assume that infants under age 1 are the only individuals eligible for the immunizations. Last, we control for aging in our model via parameter $r_{j}$ which is simply computed as $\frac{1}{|j|}$, where ∣j∣ denotes the length of age category *j* for j∈A. We let *r*_T_ = 0 since the final age category contains all individuals older than 75.

We define the force of infection for age category *j* as follows

$$\lambda^{j}\left(t\right)=\beta_{1}\left(\beta_{2}\text{cos}\left[\frac{2\pi t-\beta_{3}}{365.25/7}\right]+1\right)\;\left(\sum_{k\in A}\frac{\kappa_{k,j}}{N^{k}\left(t\right)}\sum_{l=1}^{4}\rho_{l}I_{l}^{k}\left(t\right)\right)$$ (1)

where $N^{k}\left(t\right)$ is the number of individuals in age group *k* (an alternative function with halved transmission in the off-season is included for completeness in Supplementary Text). Within the model, we multiply $\lambda^{j}$ by $\sigma_{i}$ for each $S_{i-1}$ to model a lower risk of infection after receiving the infection the respective number of times. To maintain disease dynamics, all parameters within $\lambda^{j}\left(t\right)$ must be chosen so that the function remains positive definite. Within the force of infection function, $\beta_{1}$ is the mean transmission rate, $\beta_{2}$ controls for seasonal forcing, and $\beta_{3}$ allows for possible seasonal shift to ensure simulated output corresponds with true RSV season. We control for contacts between age groups with $\kappa_{k,j}$ which is the *k,j*th entry in the US contact matrix (*22*), which relatively represents the risk of age group *k* coming in contact with age group *j*. Similarly, we control decrease of infectiousness which occurs each time individual repeatedly contracts RSV, represented by $\rho_{i}$ (*21*), which denotes the relative infectiousness of an individual who has been infected with RSV *i* times. We now define the following set of differential equations for infants under age one ($j\in \{\left[0,1\right)\}$):

$$\begin{array}{c} \frac{dS_{0}^{j}}{dt}=\left(1-\chi_{m}\right)\\ \Lambda +r_{j-1}S_{0}^{j-1}+\nu_{b}V_{b}^{j}+\nu_{m}V_{m}^{j}-\left(\sigma_{1}\lambda^{j}+\frac{3}{4}\chi_{b}+r_{j}+\mu \right)S_{0}^{j} \end{array}$$ (2)

$$\frac{dS_{1}^{j}}{dt}=r_{j-1}S_{1}^{j-1}+\gamma_{1}I_{1}^{j}-\left(\sigma_{2}\lambda^{j}+r_{j}+\mu \right)S_{1}^{j}$$ (3)

$$\frac{dS_{2}^{j}}{dt}=r_{j-1}S_{2}^{j-1}+\gamma_{2}I_{2}^{j}-\left(\sigma_{3}\lambda^{j}+r_{j}+\mu \right)S_{2}^{j}$$ (4)

$$\frac{dS_{3}^{j}}{dt}=r_{j-1}S_{3}^{j-1}+\gamma_{3}I_{3}^{j}-\left(\sigma_{4}\lambda^{j}+r_{j}+\mu \right)S_{3}^{j}$$ (5)

$$\frac{dI_{1}^{j}}{dt}=r_{j-1}I_{1}^{j-1}+\sigma_{1}\lambda^{j}S_{0}^{j}+\iota_{b}\lambda^{j}V_{b}^{j}+\iota_{m}\lambda^{j}V_{m}^{j}-\left(\gamma_{1}+r_{j}+\mu \right)I_{1}^{j}$$ (6)

$$\frac{dI_{2}^{j}}{dt}=r_{j-1}I_{2}^{j-1}+\sigma_{2}\lambda^{j}S_{1}^{j}-\left(\gamma_{2}+r_{j}+\mu \right)I_{2}^{j}$$ (7)

$$\frac{dI_{3}^{j}}{dt}=r_{j-1}I_{3}^{j-1}+\sigma_{3}\lambda^{j}S_{2}^{j}-\left(\gamma_{3}+r_{j}+\mu \right)I_{3}^{j}$$ (8)

$$\frac{dI_{4}^{j}}{dt}=r_{j-1}I_{4}^{j-1}+\sigma_{4}\lambda^{j}S_{3}^{j}-\left(\gamma_{4}+r_{j}+\mu \right)I_{4}^{j}$$ (9)

$$\frac{dR^{j}}{dt}=r_{j-1}R^{j-1}+\gamma_{4}I_{4}^{j}-\left(r_{j}+\mu \right)R^{j}$$ (10)

$$\frac{dV_{m}^{j}}{dt}=\chi_{m}\Lambda +r_{j-1}V_{m}^{j-1}-\left(\nu_{m}+\iota_{m}\lambda^{j}+r_{j}+\mu \right)V_{m}^{j}$$ (11)

$$\frac{dV_{b}^{j}}{dt}=r_{j-1}V_{b}^{j-1}+\frac{3}{4}\chi_{b}S_{0}^{j}-\left(\nu_{b}+\iota_{b}\lambda^{j}+r_{j}+\mu \right)V_{b}^{j}$$ (12)

where the model generalizes to other age categories ($j\in A\backslash \{\left[0,1\right)\}$) by setting Λ, $\chi_{b}$, and $\chi_{m}$ to zero. See Fig. 3D for model diagram and see table S8 for parameter information. To maintain demographic equilibrium, we let Λ=μ so that the total population of the system converges to 1 in the long run (see Supplementary Text). Thus, each initial condition for the system is chosen to sum up to 1 so that the age distribution of the population matches that of the actual population. After the fact, we scale the solution by the desired population size.

#### Tracking hospitalizations

We use a reporting function to track hospitalizations so that simulated output is directly comparable to empirical data. The hospitalizations for the age group *j* at time *t* is given by

$$H^{j}\left(t\right)=\lambda^{j}\left(t\right)\left(\sigma_{1}S_{0}^{j}h_{1}^{j}\theta_{j}+\sigma_{2}S_{1}^{j}h_{2}^{j}\theta_{j}+\sigma_{3}S_{2}^{j}h_{3}^{j}\theta_{j}+\sigma_{3}S_{3}^{j}h_{3}^{j}\theta_{j}\right)$$ (13)

where $h_{k}$ are given constants in (*21*) where for each age category *j*, the corresponding array entry estimates the probability of developing a lower respiratory tract infection and being hospitalized given that the individual contracted RSV for the *k*th time. We also have included θ as a vector of reporting parameters specific to our data. See Supplementary Text for details on how we adapt the hospitalization function and constants used in (*21*) to our model. We assume that this hospitalization function accounts for both hospitalization rate and the reporting rate of the hospitalization (matching the observations).

To obtain the desired disease dynamics over the given time frame, the model ran weekly for 30 years to allow for a 20-year burn-in period. The model was fit to reported RSV cases across Texas over a period of 10 years starting on 1 January 2000. Since the data were county level, the simulated hospitalizations were rescaled by the sum of the county-level population given by the 2010 census (*19*) of the counties which had reported RSV cases over the 10-year period in our dataset.

To calibrate the model, we used a Latin hypercube (LHC) random sampling method to find a set of parameters ($\beta_{1},\beta_{2},\beta_{3}$, and θ) that minimizes the Frobenius norm between the simulated and empirical numbers of hospitalizations at each time step for each age category. See Supplementary Text for details on size of parameter space searched and the refinement steps of the LHC algorithm.

#### Model initialization and simulation

The model is simulated using referenced parameter values and fitted values as in table S8. We initialize each run of the model so that the $S_{0}^{j}$ compartment is composed of the number of individuals of the *j*th age category as reported in (*31*), and we initialize $I_{1}^{j}$ to be 10,000 individuals to simulate stable outbreaks. For each run, we scale all initial conditions down so that the total population size is 1. After the simulation, we scale the population size back by the desired estimated population size. For the Texas population, we use estimates based on the 2010 US Census (*19*).

For all model runs, including fitting and all simulations, the ordinary differential equation (ODE) model was numerically integrated using weekly time steps with the ode45 integration method (using deSolve within R), beginning with a 20-year burn-in period to establish stable dynamics, followed by the desired simulation duration. For example, a 10-year simulation would be numerically solved over a period of 30 years, and only the past 10 years would be reported. To generate confidence intervals for model dynamics, we simulated the model using the top 500 of 10,000 best-performing parameter sets identified through LHC model fitting. A 95% confidence interval of the hospitalization count was then computed at each time step.

### Incorporating urbanization

We control for differences in contact patterns in urban and rural counties through parameter τ, which is based on a relative increase of 1.15 in the daycare rate seen in urban counties when compared to rural counties (*32*). τ is implemented in the model as a linear multiplier on the rows and columns of the first six age categories.

We repeatedly simulated the model on two grids of $\beta_{1}\times \tau$ and $\beta_{2}\times \tau$ values over a period of 10 years. We simulated $\beta_{1}$ (the mean transmission rate) on 0.364±0.3, $\beta_{2}$ (the seasonal forcing parameter) on 0.112±0.1, and τ (the relative urban contact rate) on 1±0.35. We fixed all the other parameters to be the best fit parameters for Texas. For each combination of parameters, we calculated the intensity based on Shannon’s entropy (*5*)

$$\text{Intensity}=1-\frac{\sum_{i=1}^{52}p_{i}\text{log}p_{i}}{\text{log}\frac{1}{52}}$$ (14)

where $p_{i}=\frac{\sum_{j}p_{i,j}}{10\sum_{i}p_{i}}$ is the average proportion of cases for week *i* and $p_{i,j}$ is the number of cases in week *i* during year *j*. This metric compares the average distribution of cases over a year to a uniform distribution of cases. The metric is equal to 1 when all cases occur in a single week and 0 when cases are uniformly distributed across the year. We also calculated the predicted proportion of under age 1 hospitalizations by taking the sum of under 1 hospitalizations over the 10-year period and dividing by the sum of hospitalizations of all ages over the 10-year period.

We also use this equation to calculate intensity in Fig. 1A. We note that smaller cities may have higher variance in case count data due to noise which may inflate the intensity metric when using observational data. This does not affect intensity calculated from simulations.

### Vaccination implementation

We model maternal vaccination through a linear relationship on the birth rate through a coverage parameter $\chi_{m}$. Note that our model assumes no innate maternal immunity in infants in the absence of vaccination. We model infant vaccination as a linear rate on the number of susceptible infants represented as $\chi_{b}$, which is the infant coverage rate. We multiply $\chi_{b}$ by $\frac{3}{4}$ to control for the fact that only infants under 9 months in the age 1 category are eligible for the infant vaccination (*7*). $\chi_{m}\;\text{and}\;\chi_{b}$ are hyperparameters used as input for each simulation depending on the desired respective coverage rates, while the the waning rates of each vaccine ($\nu_{m}$ and $\nu_{b}$) are fixed. Last, each vaccination only reduces the risk of transmission, so we model this reduced transmission as $\iota_{m}$ and $\iota_{b}$. Following (*24*), we estimate these reduced transmission rates as 1 − efficacy, where the efficacy estimates for each vaccine are as found in (*7*).

Under the current CDC guidelines, an infant is only eligible to receive one immunization. That is, if their mother received the maternal vaccine, then the infant is not eligible to receive the antibody infusion. Thus, we examined the effect of changing the ratio of infant and maternal vaccination, and we found that for year-round vaccination, applying both vaccinations at the same rate (1:1 ratio) results in the highest reduction in both under age 1 and total RSV hospitalizations, while for seasonal application, a higher coverage rate of the maternal vaccine yields the highest reduction (see Supplementary Text for details). However, the seasonal difference in percent reduction compared with equal coverage rates of the immunizations was minimal. For these reasons, we apply equal amounts of maternal and infant vaccine for all vaccination analyses.

We simulated vaccination for coverage rates varying from 0 to 100% with 0 signifying no vaccination following the CDC’s current vaccination guidelines (*7*) under which the maternal vaccine is administered from September through January and the infant immunization is administered October through March each year. We then simulated the model under the same coverage rates varying from 0 to 100% while varying the relative urban contact rate (τ) between three levels: more rural (τ=0.75), intermediate (τ=1), and more urban (τ=1.25). Under these scenarios, we calculated the proportion of hospitalizations for each age category relative to no vaccination over 10 years, and we calculated the stable maximum peak size for each age category relative to no vaccination, defined as the peak after dynamics stabilized following 15 years of vaccination implementation.

Following, we next estimated the effects of increasing the weeks of coverage of the vaccination while also increasing coverage rate. Under the CDC’s current strategy, the infant immunization is administered 26 weeks out of the year, and the maternal vaccine is administered 22 weeks out of the year (*7*). Thus, we increased the length of application of each immunization by 2 weeks at a time until each immunization was applied the entire year. We approached this symmetrically, adding a week to both ends of the interval for each additional 2 weeks of coverage. For each increase in length of coverage and each coverage rate, we computed the same metrics as before, but now with respect to dynamics for hospitalizations of infants under age 1. That is for each increase in weeks of coverage and coverage rate, we computed the total under 1 hospitalizations for 10 years after vaccination implementation relative to no vaccination, and we also computed the stable under 1 maximum peak size (the size of the peak after 15 years of vaccination implementation) relative to no vaccination.
