# Supplementary material for: Urban contact patterns shape respiratory syncytial virus epidemics with implications for vaccination
Source: Sci Adv. 2025 Nov 26;11(48):eady5457. doi: 10.1126/sciadv.ady5457 (PMC12652316; doi:10.1126/sciadv.ady5457)
Supplement: Supplementary file 1 — Supplementary Text Figs. S1 to S10 Tables S1 to S9 [file sciadv.ady5457_sm.pdf]

Science Advances
AAAS

Supplementary Materials for

Urban contact patterns shape respiratory syncytial virus epidemics with implications for vaccination

Presley Kimball *et al.*

Corresponding author: Rachel E. Baker, rebaker@brown.edu

Sci. Adv. **11**, eady5457 (2025)
DOI: 10.1126/sciadv.ady5457

This PDF file includes:

Supplementary Text
Figs. S1 to S10
Tables S1 to S9

Supplementary Text

R packages

We used the following packages for statistical and model analyses: broom v1.0.6, colorspace v2.1-1, cowplot v1.13, deSolve v1.40, doBy v4.6.22, dotwhisker v0.8.2, dplyr v1.1.4, gapminder v1.0.0, ggplot2 v3.5.1, gridBase v0.4-7, gridExtra v2.3, gridGraphics v0.5-1, haven v2.5.4, lhs v1.2.0, maps v3.4.2, mgcv v1.9-1, pals v1.9, RColorBrewer v1.1-3, readxl v1.4.3, sf v1.0-17, stargazer v5.2.3, tidyverse v2.0.0, tigris v2.1, tmap v3.3-4, and viridis v0.6.5.

Demographic Equilibrium

We provide an elementary analysis of the ODE system presented in Equations (2)-(12) in the Methods to justify the initial conditions, birth and death rates, and scaling used in modeling. First, we define the total population in the system as the sum over all states as N such that

$$N = \sum_{j \in \mathcal{A}} \sum_{s \in \mathcal{S}^j} s.$$

We now define the following set of differential equations for each $j \in \mathcal{A}$:

Note that

$$\begin{aligned}
\frac{\mathrm{d}N}{\mathrm{d}t} = \sum_{j \in \mathcal{A}} \sum_{s \in \mathcal{S}} \frac{\mathrm{d}s^j}{\mathrm{d}t} = \sum_{s \in \mathcal{S}} \frac{\mathrm{d}s^1}{\mathrm{d}t} + \sum_{j \in \mathcal{A} \setminus 1} \sum_{s \in \mathcal{S}} \frac{\mathrm{d}s^j}{\mathrm{d}t} \\
&= \Lambda - \left(\sum_{s \in \mathcal{S}} (r_1 + \mu) s^1 \right) + \left(\sum_{j \in \mathcal{A} \setminus 1} \sum_{s \in \mathcal{S}} r_{j-1} s^{j-1} - (r_j + \mu) s^j \right) \\
&= \Lambda - \left(\sum_{s \in \mathcal{S}} (r_1 + \mu) s^1 \right) + \left(\sum_{s \in \mathcal{S}} r_1 s^1 \right) - \left(\sum_{s \in \mathcal{S}} \sum_{j \in \mathcal{A} \setminus 1} \mu s^j \right) \\
&= \Lambda - \left(\sum_{s \in \mathcal{S}} \mu s^1 \right) - \left(\sum_{s \in \mathcal{S}} \sum_{j \in \mathcal{A} \setminus 1} \mu s^j \right) \\
&= \Lambda - \mu \left(\sum_{j \in \mathcal{A}} \sum_{s \in \mathcal{S}} s^j \right) \\
&= \Lambda - \mu N.
\end{aligned}$$

We analytically have a solution to this ODE:

$$N(t) = \frac{\Lambda}{\mu} + \left(N(0) - \frac{\Lambda}{\mu}\right) e^{-\mu t}. \tag{S1}$$

Note that as consequence, $\lim_{t\to\infty} N(t) = \frac{\Lambda}{\mu}$. Hence, choosing $\Lambda = \mu$ causes $N(t)$ to approach 1 for all initial conditions. We therefore rescale all solutions by the desired population size so that simulations on the same order of observed data.

Tracking Hospitalizations

The hospitalization function used in our model follows from (*21*). The model uses published literature to estimate the probability of developing a lower respiratory tract infection due to an RSV infection for a partition of pediatric age groups. The authors then estimate the proportion of RSV infections that result in hospitalization for each age group by scalar multiplication of the probability of developing a lower respiratory tract infection. For our purposes, we consolidate these estimates into the vectors $\mathbf{h_1}$, $\mathbf{h_2}$, and $\mathbf{h_3}$, where each entry represents the relative risk of hospitalization for the corresponding age group for the first, second, and third/fourth infections, respectively.

These estimates of hospitalization rates assume that during the first six months of an infant's life, the infant has the highest risk of developing a lower respiratory tract infection given an RSV infection. During the subsequent six months (months 6-11), the risk for a lower respiratory tract infection is reduced by almost half, and in the following year for 1-2 year olds, the same risk drops by more than 10%. Similarly when examining the proportion of lower respiratory infections caused by RSV that result in hospitalization, the authors estimate the highest risk occurs in infants under 3 months, followed by a gradual decline with age. See Zheng *et al.* (*21*) for details and exact numerical estimates. In our model, we derive a single hospitalization rate for infants under one year old by averaging across all subcategories.

Additionally, the hospitalization rates decrease with the number of times an individual contracts the disease. We assume that the relative risk of infection declines with repeated infections, represented by $\sigma_1 \geq \sigma_2 \geq \sigma_3$. Similarly, the proportion of RSV infections leading to hospitalization decreases with repeated infections, represented by $\mathbf{h_1} \geq \mathbf{h_2} \geq \mathbf{h_3}$, where the inequality holds entry-wise.

Note our reporting differs from (*21*) since we multiply $\mathbf{h_1}, \mathbf{h_2}$ by $\boldsymbol{\theta}$. We add $\boldsymbol{\theta}$ as a fitted parameter since we are fitting to a different data and thus the average reporting rate is expected to change.

Model Fitting

As noted in the main text, we use a Latin Hyper Cube algorithm to fit $(\beta_1, \beta_2, \beta_3, \boldsymbol{\theta})$. To limit the dimensionality issue that comes with random-sampling, we first conducted a LHC random sample of size 1,000 for (β_1, β_2, and β_3 and assumed $\boldsymbol{\theta} = \vec{1}$. This makes the assumption that all hospitalization parameters as reported by (*21*) were optimal. This initial LHC random sample searched for $\beta_1 \in [0, 4], \beta_2 \in [0, 1]$, and $\beta_3 \in \left[\frac{\pi}{3}\frac{365.25}{7}, \frac{2\pi}{3}\frac{365.25}{7}\right]$. β_1 was on this range by empirical estimation, β_2 was on this range so that the force of infection function λ remained positive definite, and β_3 was on this range based on seasonal estimates given in (*21*). After the first LHC random sample was conducted, the top 50 parameter sets were collected, and the LHC random sample was repeated over β_1, β_2, and β_3 using the ranges of each parameter in the top 50 parameter sets.

For the third round of LHC fitting, we fixed β_1, β_2, and β_3 to be the values which minimized the loss in the previous LHC iteration and conducted LHC random sample parameter estimation for θ_j for $j = \{1, 2, ..., 6\}$. As found in preliminary analysis, we are aiming to focus on age differences below age 10 which coincides with the chosen values of rescaling. In the final LHC search, we created an LHC random sample of 10,000 parameter sets over the entire parameter space (β_1, β_2, β_3, θ_1, θ_2, θ_3, θ_4, θ_5, and θ_6). For each parameter β_i and θ_j, the search was done over the range of the respective parameter given from the top 2.5% and 5% of parameters of each last LHC random sample.

Nonlinear Effects on RSV Age Distribution Shape Parameters

We investigate possible nonlinear effects of log population density and average specific humidity on the RSV age distribution shape parameters using generalized additive models (GAMs) in fig. S4. We first fit additive smooth models (limited to 5 basis functions) predicting for parameters b and k as in Fig. 1. Both log population density (b: edf = 3.246, F = 20.81, $p < 2\mathrm{e}^{-16}$; k: edf = 3.185, F = 20.660, $p < 2\mathrm{e}^{-16}$) and average specific humidity (b: edf = 3.943, F = 10.93, $p < 2\mathrm{e}^{-16}$; k: edf = 3.936, F = 9.997, $p < 2\mathrm{e}^{-16}$) had significant nonlinear effects. However, as demonstrated in the splines S4, the effect due to average specific humidity fluctuates around zero which may indicate

overfitting over a true nonlinear effect.

To explore the nonlinear relationship more thoroughly, we fit additional smooth bivariate GAMs using the same two predictors. Analysis of deviance revealed that bivariate smooth GAM provided a better fit than the additive smooth GAM for both shape parameters (b: F = 57.484, p = 5.554e^{11}; k: F = 114.24, p = 1.211e^{-10}). The smooth bivariate effect was also found to be statistically significant for both shape parameters (b: F = 18.85, p < 2e^{-16}; k: F = 18.25, p < 2e^{-16}), with the shapes of the effects shown in (b) and (c) of fig. S4A and B. As demonstrated in the heat maps of the estimated partial effects, the level sets run horizontally, indicating that the partial effects are notably more sensitive to log population density than to average specific humidity. The surface plot further illustrates that the estimated partial effect on the shape parameter is strongly influenced by log population density over average specific humidity.

Application of Maternal versus Infant Immunizations

As noted in the main text, current CDC guidelines mandate that an infant is only eligible to receive either the maternal immunization through their parent or the infant immunization. Therefore, we compared the effects of different applications of each vaccine at the population-level. To do this, we linearly computed individual coverage rates for the maternal and infant vaccine based on the ratio. That is,

$$\chi_{\text{maternal}} = m \cdot \chi_{\text{total}} \tag{S2}$$

$$\chi_{\text{infant}} = (1 - m) \cdot \chi_{\text{total}} \tag{S3}$$

where χ_{total}, χ_{maternal}, and χ_{infant} are the total, maternal, and infant coverage rates, respectively, and m is the percent of vaccinations which are maternal. We then simulated the model under coverage rates ranging from 0 to 1 and $m \in \{1, \frac{2}{3}, \frac{1}{2}, \frac{1}{3}, 0\}$. We repeated this process for both CDC timing and year round vaccination.

In figure S10, we observe how the number of RSV hospitalizations changes with coverage rate as we vary the ratio of each vaccination. In particular, we computed these differences in reduction in table S9. We found that an equal ratio of maternal and infant vaccination resulted in the highest reduction of both under age 1 and total RSV hospitalizations when the immunizations are applied year-round. When the immunizations are applied seasonally, a higher ratio of maternal vaccine

application results in a slightly higher percent reduction in total and under age 1 RSV cases with a $< 0.5\%$ and $< 0.13\%$ difference in percent reduction, respectively. This is due to the waning immunity of the maternal and infant immunizations, with since the immunity resulting from the maternal vaccine is longer than that of the infant immunization. For our analyses, we proceeded with equal coverage rate for the two immunizations, as this combination yielded the largest reduction when applied year-round, and for seasonal application, the difference compared with the optimal scenario was minimal.

Off-Season Transmission Correction

One limitation of our model was the inability to fully capture the depth of the trough of hospitalizations during RSV off-seasons. To attempt to correct this, we modified our force of infection function to be

$$\lambda^j(t) = \begin{cases} \frac{1}{2}\lambda^j_{\text{prev}}(t), & \text{if } t \in [21, 25) \\ \lambda^j_{\text{prev}}(t), & \text{else} \end{cases} \tag{S4}$$

where $\lambda^j_{\text{prev}}(t)$ is the same force of infection as defined in Equation (1). The interval $[21, 25)$ approximately corresponds to the last two weeks of May and first two weeks of June. We then simulated the model using the best fit parameters for Texas with and without vaccination. For vaccination, we implement the CDC's recommended timing as well as year-round vaccination for coverage rates of 0.5 and 0.9. figure S9 contains these results. We find similar dynamics as found in the main text without the correction.

Shift in Outbreak Timing Due to Vaccination

To understand the changes in stable maximum peak size due to increased vaccination coverage and weeks of coverage as seen in Fig. 4, we investigated how increased vaccination affects outbreak timing. We repeated the simulations used to generate Fig. 4C and D, but this time measured the timing of the annual maximum peak (relative to no vaccination) during the 10th year of implementation, when vaccination dynamics have sufficiently stabilized. Results are shown in fig. S8. We find that under seasonal vaccination, increased coverage rate causes a positive forward shift in outbreak timing. This is because at lower coverage rates a portion of transmission occurs while

vaccination is underway: at higher coverage rates transmission is paused during the vaccination season. Under our model, urban environments have a higher force of infection on average: in this case, high values of vaccination coverage force outbreaks to occur outside of the vaccination window. In contrast, rural areas have a lower force of infection on average which causes outbreak timing to be less sensitive to vaccination.

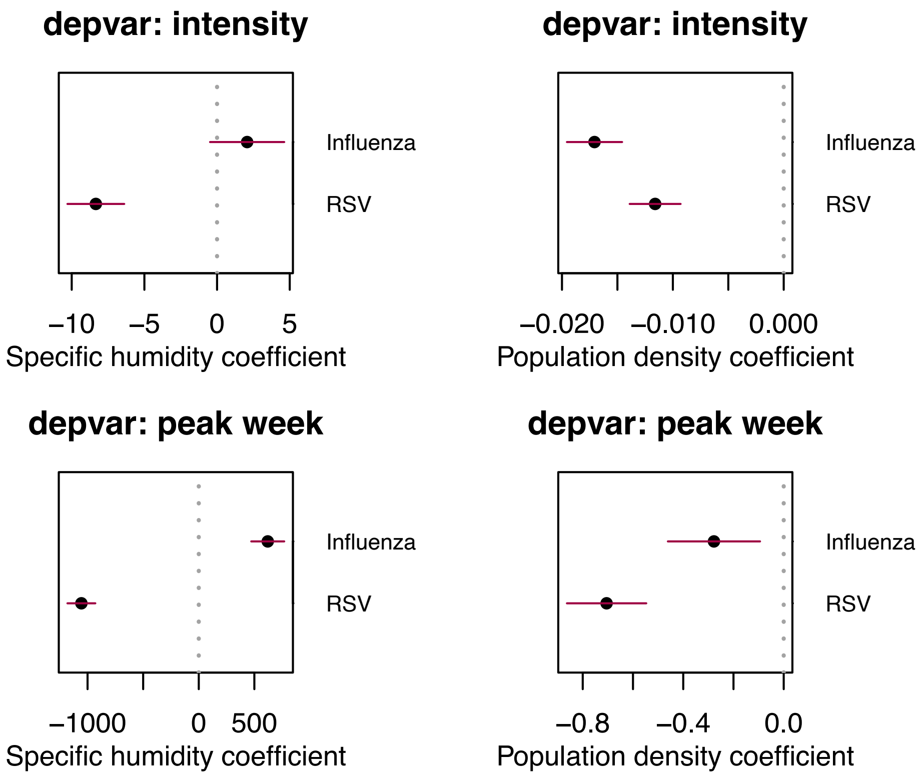

Figure S1: **OLS regression coefficients.** Point estimate with confidence intervals for OLS regression of climate (mean specific humidity (kg/kg) and log population density ($capita/km^2$) on epidemic intensity and peak timing for RSV and influenza. These regression results show the statistical association behind Fig. 1A.

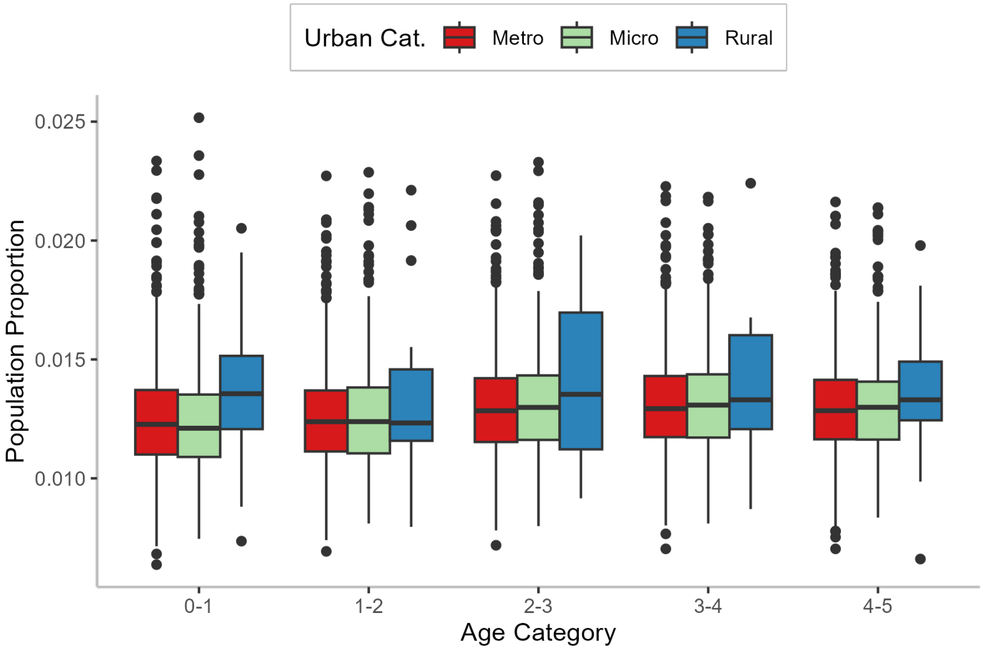

Figure S2: **Under 5 age distribution across United States conditioned on urbanization category.** Differences in age distribution are not statistically significant for ages under 5 when conditioned on urbanization category (see table S4 for exact ANOVA values).

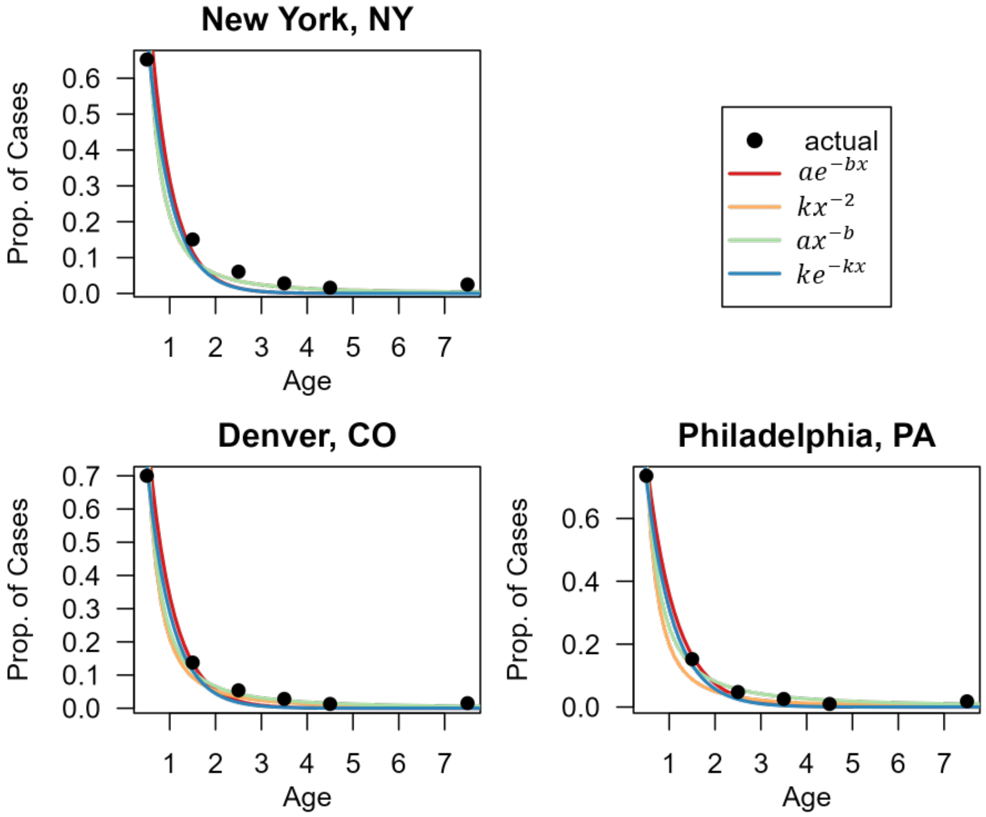

Figure S3: **Examples of fitted parameterized curves of the RSV age distribution.** We provide three example locations with each of the four tested curves fitted to demonstrate how each model compares to data.

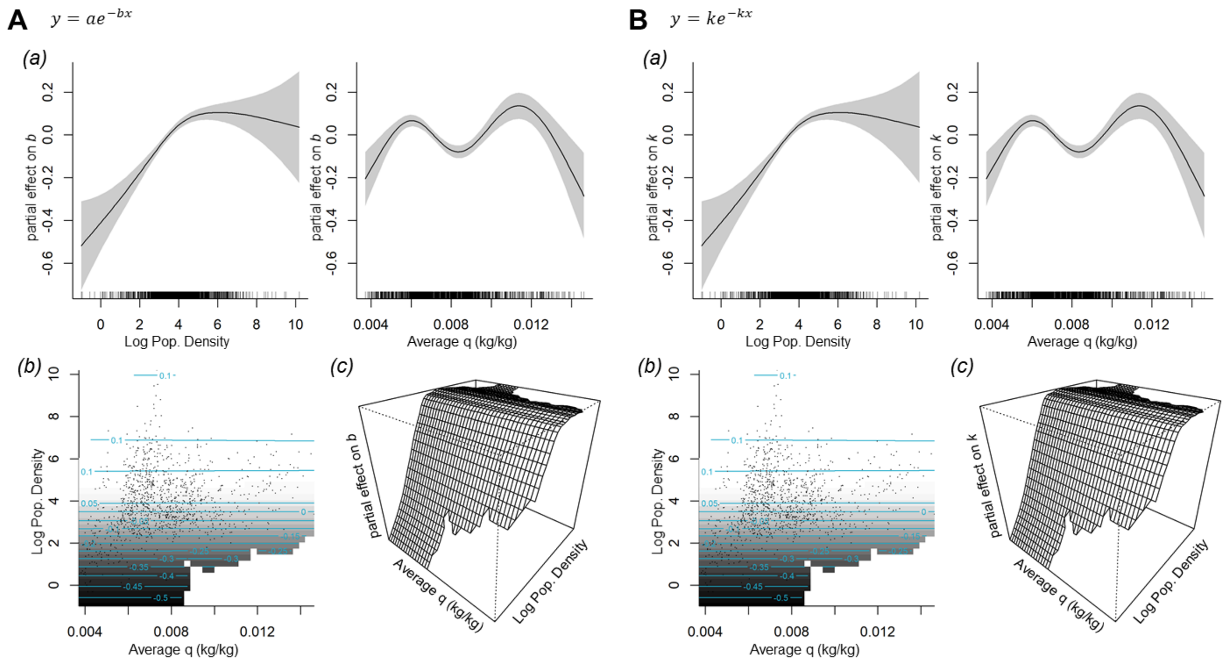

Figure S4: **Generalized additive models (GAMs) suggest shape parameters of RSV age-distribution in Fig. 1 are more strongly influenced by log population density than average specific humidity.** GAM results of using log population density and average specific humidity, q, for predicting shape parameters (A) b from fitted $y = ae^{-bx}$ and (B) k from fitted $y = ke^{-kx}$. (a) Resulting splines of fitted additive smooth GAM utilizing log population density and average specific humidity as predictors. (b)/(c) Heat map/surface plot of the partial effect on respective shape parameter estimated by bivariate smooth GAM, using the same predictors as (a).

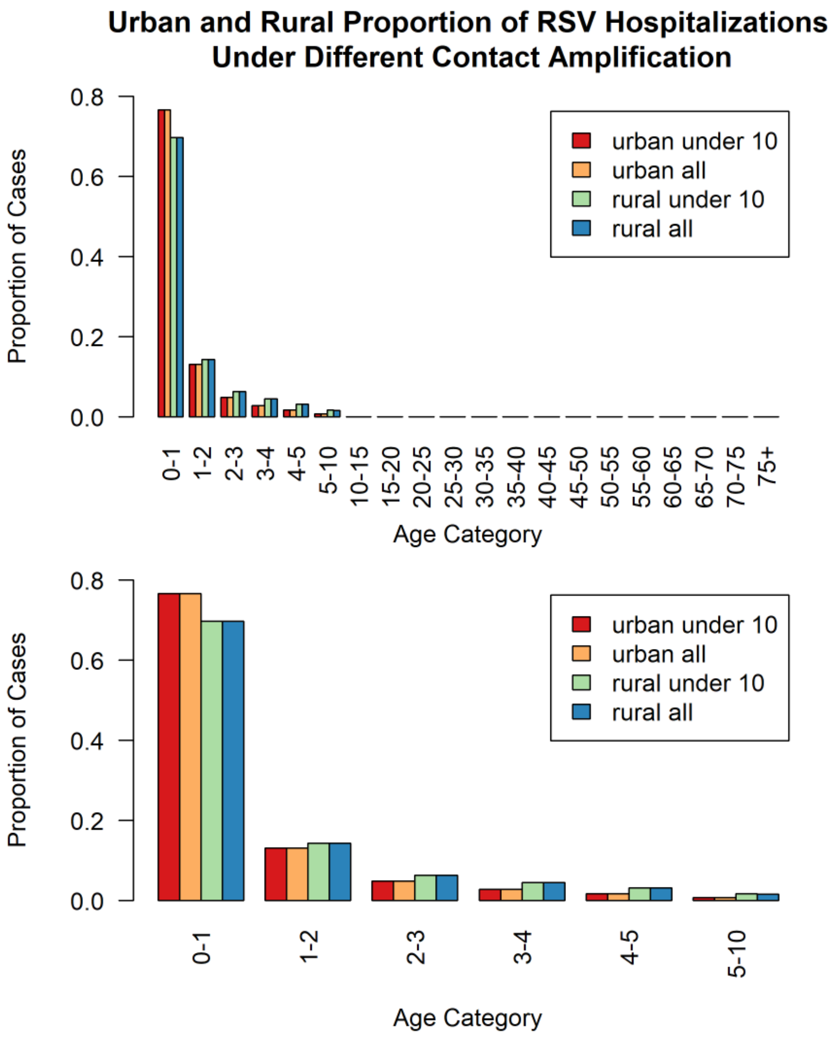

Figure S5: **Applying relative urban contact rate amplification (τ) on subset of age categories yields almost same effect as applying on all age categories.** Top plot displays entire age distribution of RSV hospitalizations under four scenarios: applying contact rate amplifier τ to just age-categories under 10 and applying to all age categories, both in urban and rural environments. Bottom plot displays the same four distributions for the first six age categories.

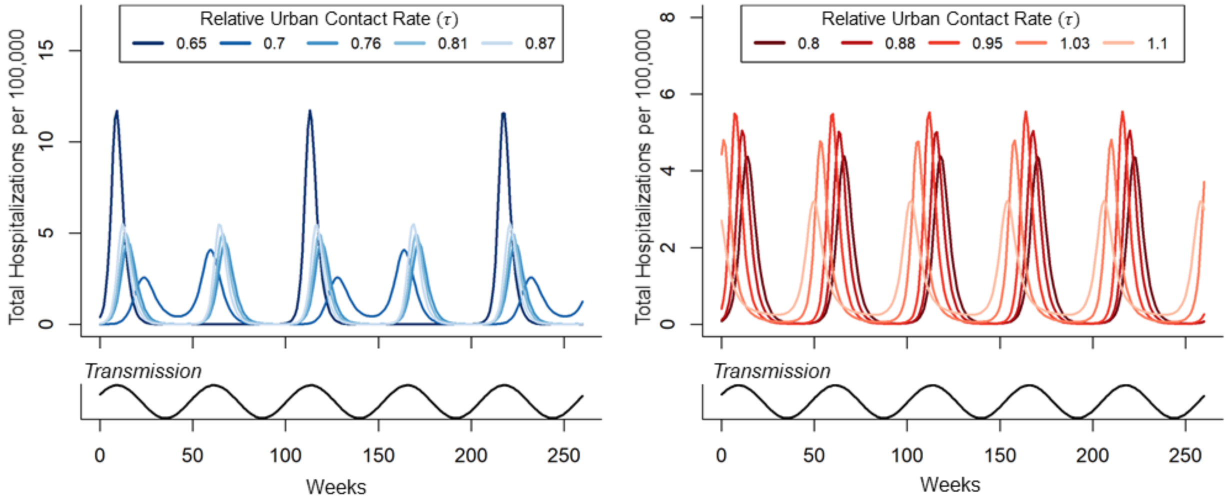

Figure S6: **The non-linear relationship between relative urban contact rate (τ) and intensity (Fig 3C) are due to bifurcations and dynamical resonance.** Left plot shows dynamics for high seasonal forcing ($\beta_2 = 0.2$) and various low values of τ which correspond to bifurcation on a horizontal slice of top left of first plot in Fig. 3C. Right plot displays dynamics over horizontal slice for moderately high seasonal forcing ($\beta_2 = 0.16$) over intermediate τ values demonstrating dynamical resonance with the seasonal transmission function.

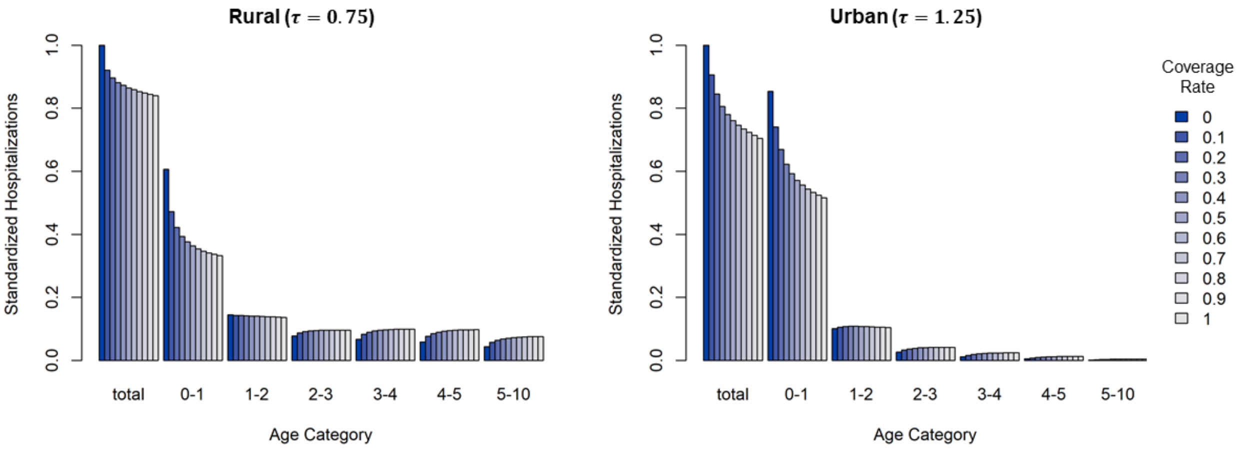

Figure S7: **Higher immunization coverage rates lead to steeper decline in RSV hospitalizations in urban communities as found in Fig. 4C.** Bar plots show that in more urban environments with a higher relative urban contact rate τ results in a steeper decline in total and under age one RSV hospitalizations when compared to rural environments.

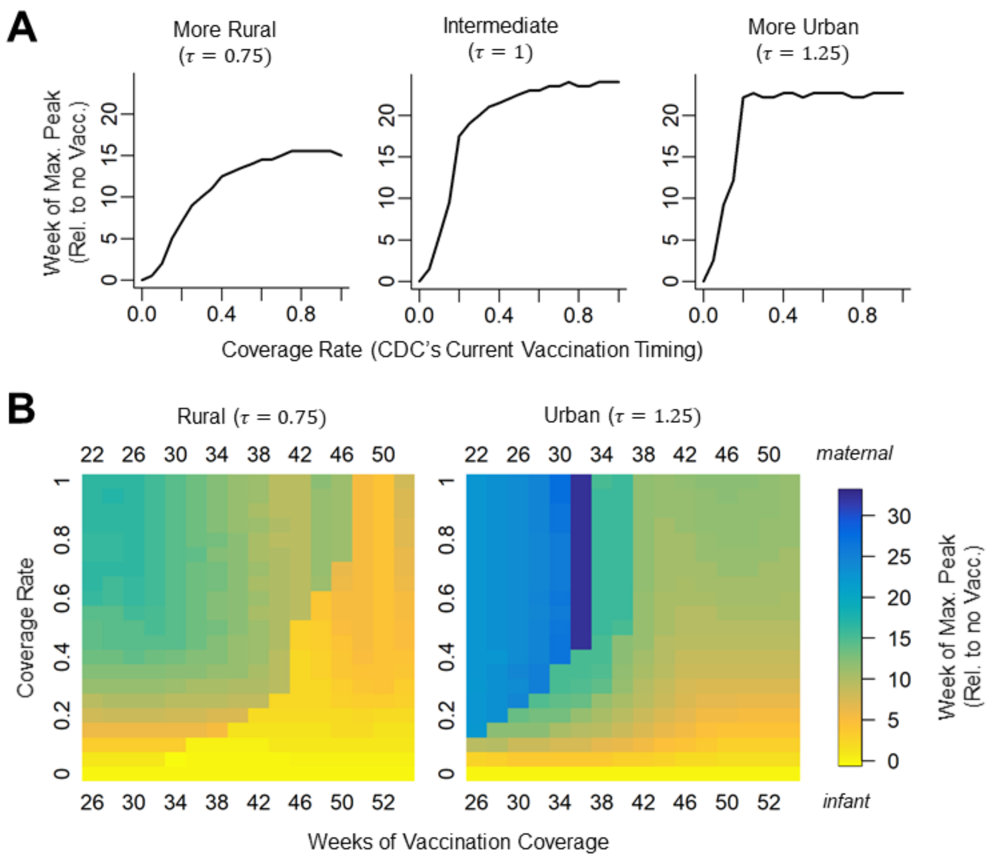

Figure S8: **Increased vaccination causes shift in timing of annual outbreaks.** (**A**) Under CDC seasonal vaccination, increased coverage rate causes timing of vaccination to shift forward monotonically (non-monotonic fluctuation on tails is due to discretization of numerical integration). Higher relative urban contact rate (τ) results in a faster shift with respect to contact rate, with the shift eventually plateauing in more urban areas as outbreak has completely shifted outside of the vaccination window for high coverage rates. (**B**) Generated heat maps demonstrate nonlinear effects on timing of largest outbreak due to higher coverage rate and increased weeks of vaccination coverage.

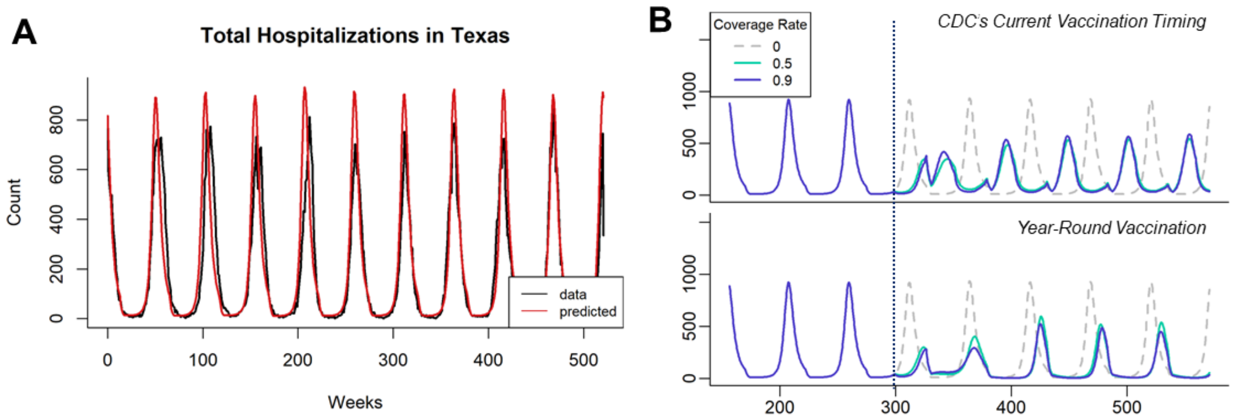

Figure S9: **Model with off-season transmission correction provides similar vaccination dynamics.** (**A**) Total hospitalizations in Texas compared against model with transmission halved during week 21 until week 25. (**B**) Model dynamics with transmission halved during week 21 until week 25 provide similar outbreak dynamics (after stabilizing) as in Figure 4 B.

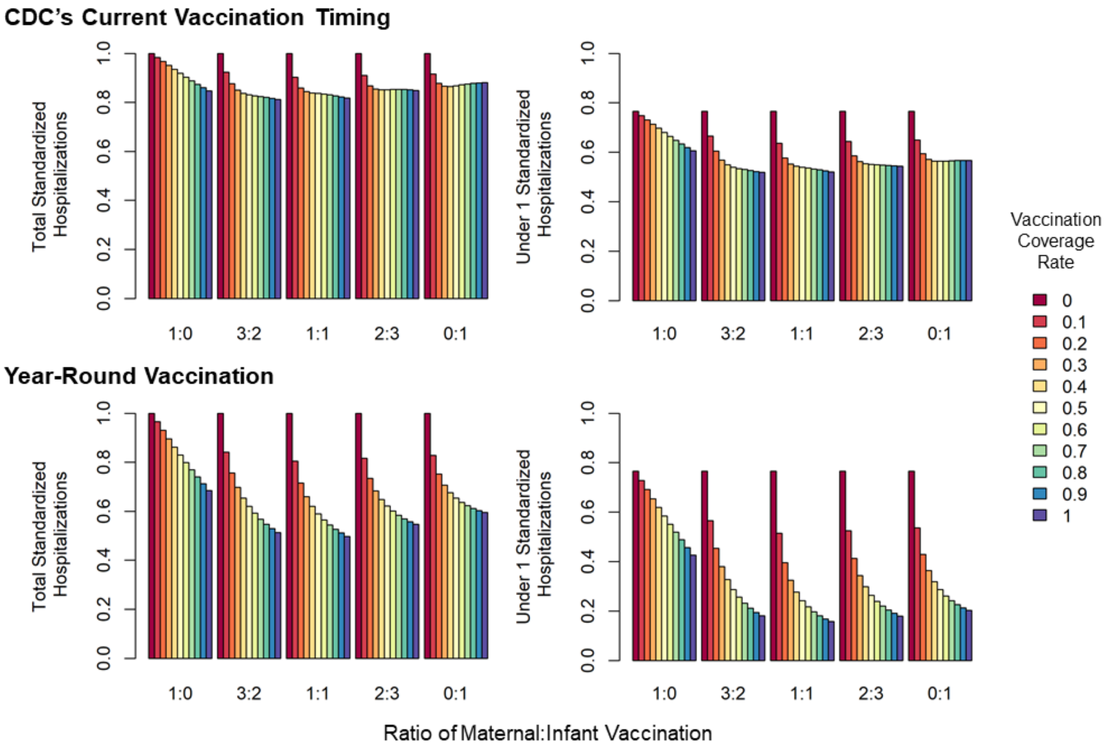

Figure S10: **Implementing an equal ratio of maternal and infant vaccination results in the largest percent reduction in hospitalizations under year-round vaccination and second largest percent reduction in seasonal vaccination.** Under seasonal vaccination (top: CDC's Current Vaccination Timing) a 3:2 ratio of maternal and infant immunization results in the highest percent reduction in total (under 1) hospitalizations, with equal allocation producing a reduction only < 0.5% (< 0.13%) lower. Year-round vaccination (bottom), total and under age 1 hospitalizations both have the lowest amount of cases under equal application of the maternal and infant vaccines, as well as with full coverage rate. See table S9 for reduction values.

Table S1: **Regression of urban category (metro/micro/rural) on under 1 RSV proportion.** Note that due to perfect multicollinearity in the categorical predictor variable ”metro” is omitted.

	Dependent variable:	
	Proportion RSV hospitalizations in 0-1 age class	
	(1)	(2)
Urban category: Micro	-0.038^{***}	-0.040^{***}
	(0.005)	(0.005)
Urban category: Rural	-0.152^{***}	-0.154^{***}
	(0.018)	(0.018)
Population size		−-4.631e-09
		(0.000)
Constant	0.760^{***}	0.762^{***}
	(0.003)	(0.003)
Observations	1,118	1,118
R^2	0.107	0.108
Adjusted R^2	0.106	0.106
Residual Std. Error	0.072 (df = 1115)	0.072 (df = 1114)
F Statistic	66.886^{***} (df = 2; 1115)	44.942^{***} (df = 3; 1114)
Note:		*p<0.1; **p<0.05; ***p<0.01

Table S2: **Quasibinomial GLM regression.** Odds ratios (OR) for the proportion of RSV hospitalizations by age group and urbanization category interacted. Reference (intercept) category is age <1 year and metro.

Predictor	OR	95% Confidence Interval	p-value
Intercept (Age <1 yr, Metro)	3.19	3.14 – 3.24	< 0.001
Age 1–2 yr	0.054	0.053 – 0.056	< 0.001
Age 2–3 yr	0.016	0.016 – 0.017	< 0.001
Age 3–4 yr	0.007	0.0069 – 0.0073	< 0.001
Age 4–5 yr	0.003	0.0029 – 0.0031	< 0.001
Age 5–10 yr	0.0036	0.0034 – 0.0038	< 0.001
Micro vs Metro (Age <1 yr)	0.81	0.78 – 0.84	< 0.001
Rural vs Metro (Age <1 yr)	0.54	0.44 – 0.66	< 0.001
Age 1–2 yr × Micro	1.62	1.54 – 1.71	< 0.001
Age 2–3 yr × Micro	1.46	1.36 – 1.56	< 0.001
Age 3–4 yr × Micro	1.25	1.11 – 1.41	< 0.001
Age 4–5 yr × Micro	1.10	0.92 – 1.32	0.279
Age 5–10 yr × Micro	0.80	0.67 – 0.96	0.019
Age 1–2 yr × Rural	2.85	2.07 – 3.92	< 0.001
Age 2–3 yr × Rural	3.36	2.23 – 5.05	< 0.001
Age 3–4 yr × Rural	3.45	1.97 – 6.05	< 0.001
Age 4–5 yr × Rural	2.66	1.08 – 6.54	0.033
Age 5–10 yr × Rural	2.88	1.31 – 6.33	0.008

Table S3: **The differences in the RSV age distribution due to urbanization are statistically significant.** ANOVA column gives the significance of the the ANOVA test for each age group across urbanization categories. The last three columns present the pairwise p-values computed using Tukey-HSD.

Age Category	ANOVA	Metro–Micro	Metro–Rural	Micro–Rural
0–1	$< 2e{-}16$*	0*	0*	0*
1–2	$< 2e{-}16$*	0*	1e−7*	0*
2–3	4.31e−15*	0*	0*	5.78e−5*
3–4	2.79e−6*	0.0391*	1.04e−5*	2.06e−4*
4–5	0.0347*	0.959	0.0258*	0.0339*
5–10	5.64e−7*	0.672	6e−7*	2e−7*

Table S4: **The differences in the population-level age distribution due to urbanization are not statistically significant for ages under 10.** Presented values result from ANOVA test on proportion of population of six age categories for ages under 10 when conditioned by urbanization category (which results in 2 degrees of freedom). See Figure S2 for visualization of distributions for ages under 5.

Age Category	Sum of Squares	Mean Square	F-statistic	p-value
0–1	0.000019	$9.394e-06$	1.682	0.186
1–2	0.000027	$1.341e-05$	2.614	0.0737
2–3	0.000018	$9.016e-06$	1.766	0.171
3–4	0.000017	$8.63e-06$	1.805	0.165
4–5	0.000012	$5.922e-06$	1.321	0.267
5–10	0.00009	$4.729e-05$	0.513	0.599

Table S5: **The differences in the measures of the rate of change in the RSV age distribution due to urbanization are statistically significant.** ANOVA gives the significance of the the ANOVA test generated across all urbanization categories per each age group. The last three columns present the pairwise p-values computed using Tukey-HSD.

model	ANOVA	Metro–Micro	Metro–Rural	Micro–Rural
b: $y = ae^{-bx}$	< 2e−16*	0*	0*	0.0001149*
k: $y = ke^{-kx}$	< 2e−16*	0*	0*	0*

Table S6: **Regression shows log of population density and average humidity are statistically significant predictors of the shape of the RSV distribution.** Mixed and simple regression was computed with log of population density and average humidity as predictors for the b in the $y = ae^{-bx}$ model.

	Dependent variable:		
	b from fitted $y = ae^{-bx}$		
	(1)	(2)	(3)
Log Pop. Density	0.069^{***}		0.067^{***}
	(0.007)		(0.007)
Avg. Humidity		19.810^{***}	7.307
		(5.330)	(5.312)
Constant	1.275^{***}	1.397^{***}	1.229^{***}
	(0.029)	(0.042)	(0.044)
Observations	1,117	1,117	1,117
R^2	0.081	0.012	0.083
Adjusted R^2	0.081	0.011	0.081
Residual Std. Error	0.340 (df = 1115)	0.352 (df = 1115)	0.340 (df = 1114)
F Statistic	98.810^{***} (df = 1; 1115)	13.813^{***} (df = 1; 1115)	50.390^{***} (df = 2; 1114)
Note:			$^{*}p<0.1$; $^{**}p<0.05$; $^{***}p<0.01$

Table S7: **Regression shows log of population density and average humidity are statistically significant predictors of the shape of the RSV distribution.** Mixed and simple regression was computed with log of population density and average humidity as predictors for the k in the $y = ke^{-kx}$ model.

	Dependent variable:		
	k from fitted $y = ke^{-kx}$		
	(1)	(2)	(3)
Log Pop. Density	0.067***		0.064***
	(0.006)		(0.006)
Avg. Humidity		20.528***	8.550*
		(4.632)	(4.578)
Constant	1.309***	1.416***	1.255***
	(0.025)	(0.036)	(0.038)
Observations	1,117	1,117	1,117
R^2	0.100	0.017	0.103
Adjusted R^2	0.099	0.016	0.101
Residual Std. Error	0.293 (df = 1115)	0.306 (df = 1115)	0.293 (df = 1114)
F Statistic	123.913*** (df = 1; 1115)	19.643*** (df = 1; 1115)	63.839*** (df = 2; 1114)

Note: *p<0.1; **p<0.05; ***p<0.01

Table S8: **Parameter information for the SIR model.** We provide the symbolic parameters, their meanings, values, and sources. The lowest loss parameters for the force of transmission function (λ^j) and hospitalization reporting parameter fitted to Texas data are also presented.

variable	meaning	value	sources
Λ	birth rate	$\approx \frac{15.34\,\text{births}}{1000\,\text{people}}$	(*19, 30*)
μ	death rate	Λ	
λ^j	force of infection for $j \in \mathcal{A}$	calculated, dynamic	
γ_i	recovery rate or I_i	[1/10,1/7,1/5,1/5] $\frac{1}{\text{day}}$	(*21*)
ρ_i	infectiousness of I_i	[1,0.75,0.51,0.51]	(*21*)
σ_i	risk of infection for S_{i-1}	[1,0.76,0.6,0.4]	(*21*)
r_j	age rate for age category $j \in \mathcal{A}$	$r_j = \begin{cases} 1/\lvert j\rvert, & \text{if } j \notin \{0, T\} \\ 0, & \text{else} \end{cases}$	NA
$\kappa_{i,j}$	entry in contact matrix corresponding to interaction between i and j age groups	see U.S. contact matrix in (*22*)	(*22*)
h_i^j	prob. of hospitalization for ith infection	for $\mathbf{h_1}$ and $\mathbf{h_2}$ see (*21*); $\mathbf{h_3} = \mathbf{0}$	(*21*)
$\boldsymbol{\theta}$	vector containing fitted hospitalization reporting parameters for each age category	best fit: [0.748, 1.281, 1.755, 3.196, 5.95, 11.399, 1, ..., 1]	NA
β_1	fitted mean transmission rate	best fit: 0.364	NA
β_2	fitted seasonal forcing	best fit: 0.113	NA
β_3	fitted seasonal shift	best fit: 57.8	NA
τ	under 5 daycare amplification parameter	urban: 1, rural: $\frac{23.4}{26.9}$	(*32*)
χ_m	coverage rate of maternal vaccine	varies	NA
χ_b	coverage rate of infant immunization	varies	NA
ν_m	waning rate of maternal vaccine	(6 months)$^{-1}$	(*7*)
ν_b	waning rate of infant immunization	(5 months)$^{-1}$	(*7*)
ι_m	risk of infection under maternal vaccine	(1-0.57)	(*7*)
ι_b	risk of infection under infant immunization	(1-0.80)	(*7*)

Table S9: **Applying equal coverage of maternal and infant immunization year round results in the highest reduction in RSV cases, and applying equal coverage provides the second highest reduction in RSV cases when applying seasonally (CDC timing).** We show percent reduction following the CDC's current vaccination timing and full year round coverage. Each column represents the percent reduction in total or under age 1 cases in RSV for a fixed ratio of maternal:infant vaccination coverage.

Vacc. Timing	Ages	1:0	3:2	1:1	2:3	0:1
CDC	Total	0.1532010	0.1876417	0.1827187	0.1509452	0.1188750
	Under 1	0.2080423	0.3214837	0.3202466	0.2902054	0.2596825
Year Round	Total	0.3159287	0.4871798	0.5024626	0.4534836	0.4047971
	Under 1	0.4425721	0.7631915	0.7922820	0.7634317	0.7349743
